# Chicken Liver–Associated Outbreaks of Campylobacteriosis and Salmonellosis, United States, 2000–2016: Identifying Opportunities for Prevention

**DOI:** 10.1089/fpd.2018.2489

**Published:** 2018-11-13

**Authors:** William A. Lanier, Kis Robertson Hale, Aimee L. Geissler, Daniel Dewey-Mattia

**Affiliations:** ^1^ U.S. Public Health Service Commissioned Corps, Rockville, Maryland.; ^2^ Food Safety and Inspection Service, U.S. Department of Agriculture, Washington, District of Columbia.; ^3^ Centers for Disease Control and Prevention, U.S. Department of Health and Human Services, Atlanta, Georgia.

**Keywords:** liver, chicken, infectious disease outbreaks, foodborne diseases, *Campylobacter*, *Salmonella*, prevention and control, cooking

## Abstract

Chicken liver has been implicated in several reported U.S. illness outbreaks, probably caused by inadequate cooking and pathogen contamination. To identify commonalities among these outbreaks that could represent targets for prevention, we describe chicken liver–associated U.S. outbreaks during 2000–2016 reported to the Food Safety and Inspection Service, to the Centers for Disease Control and Prevention, and in published literature. We identified 28 outbreaks (23 [82.1%] were campylobacteriosis only, 3 [10.7%] were salmonellosis only, and 2 [7.1%] were caused by both pathogens), with 18 (64.3%) occurring during 2014–2016. Common outbreak features included blended chicken liver dishes (e.g., pâté; 24 [85.7%]), inadequate cooking (26 [92.8%]), and preparation in foodservice settings (e.g., sit-down restaurants; 25 [89.3%]). The increasing frequency of reported outbreaks highlights chicken liver as an important food safety problem. Public health partners should collaborate on prevention measures, including education on proper foodservice preparation of blended chicken liver dishes.

## Introduction

*C**ampylobacter* and *Salmonella* are the estimated leading causes of bacterial foodborne infections in the United States (Scallan *et al.*, 2011). Infections with these pathogens are rarely fatal but can be associated with severe gastrointestinal symptoms, sometimes requiring hospitalization, and can lead to postinfectious complications, including reactive arthritis, irritable bowel syndrome, and Guillain–Barré syndrome (Nyati and Nyati, 2013; Scallan *et al.*, 2015). Exposure to chicken and other poultry products has been identified as both a common source of campylobacteriosis and salmonellosis outbreaks (Chai *et al.*, 2017) and as a risk factor for sporadic infection with these pathogens (Friedman *et al.*, 2004; Kassenborg *et al.*, 2004; Kimura *et al.*, 2004; Fullerton *et al.*, 2007).

Chicken liver has been recognized as an important vehicle for foodborne infections (Geissler *et al.*, 2017). Like other chicken parts, chicken liver is regulated by the Food Safety and Inspection Service (FSIS). Methods of preparing chicken liver for consumption are numerous and include frying and blending (e.g., for pâté). Recent outbreaks of campylobacteriosis and salmonellosis in the United States have been linked to chicken liver (Tompkins *et al.*, 2013; Hanson *et al.*, 2014; Scott *et al.*, 2015; Glashower *et al.*, 2017). Such outbreaks have also occurred in other countries; reviews of chicken liver–associated outbreaks in the United Kingdom (Little *et al.*, 2010), Australia (Merritt *et al.*, 2011), and New Zealand (NZMPI, 2007, 2013) have been published. These outbreaks may, in large part, be explained by the interplay of two factors: inadequate cooking and pathogen contamination.

FSIS and the Food and Drug Administration (FDA) recommend cooking poultry products to an internal temperature of 165°F (FDA, 2013; FSIS, 2015). However, some food writers recommend using livers that have not been fully cooked (e.g., “still rosy pink inside”) when preparing chicken liver dishes (The Dallas Morning News, 2013; Los Angeles Times, 2015). Recipes that call for the use of partially cooked chicken liver are readily available (The New York Times Company, 2018; Food Network, 2018).

Cooking to recommended internal temperatures is especially important for foods known to contain pathogens. Like other chicken products, chicken liver has been found to be contaminated with *Campylobacter* (Cox *et al.*, 2009; Noormohamed and Fakhr, 2012; Strachan *et al.*, 2012) and *Salmonella* (Zdragas *et al.*, 2012; Abd-Elghany *et al.*, 2015). Of particular concern is the evidence that pathogens can exist in internal chicken liver tissues. Among several studies, *Campylobacter* was recovered from the internal tissues of 10–90% of chicken livers tested after the external surface had been sterilized (Barot *et al.*, 1983; Boukraa *et al.*, 1991; Baumgartner *et al.*, 1995; Whyte *et al.*, 2006; Firlieyanti *et al.*, 2016). In addition, in studies involving specific pathogen–free chickens in experimental conditions, livers tested after oral inoculation yielded *Campylobacter* (Sanyal *et al.*, 1984; Knudsen *et al.*, 2006; Chaloner *et al.*, 2014) and *Salmonella* (Borsoi *et al.*, 2009; He *et al.*, 2010; Gast *et al.*, 2013). Pathogens are thought to spread from the gastrointestinal tract to the liver through the biliary, lymphatic, or vascular systems, although the exact route is unclear (Boukraa *et al.*, 1991; Whyte *et al.*, 2006; He *et al.*, 2010; Chaloner *et al.*, 2014; Firlieyanti *et al.*, 2016).

Consumption of inadequately cooked chicken livers contaminated with pathogens can lead to illnesses and outbreaks. To better understand the characteristics of such outbreaks to identify opportunities for prevention, we reviewed and described chicken liver–associated outbreaks in the United States during 2000–2016.

## Materials and Methods

We reviewed outbreaks reported to FSIS and the Foodborne Disease Outbreak Surveillance System (FDOSS) of the Centers for Disease Control and Prevention (CDC, 2017a) and in published literature to identify outbreaks of illness associated with chicken liver during 2000–2016 in the United States. An outbreak was defined as the occurrence of two or more cases of similar illness resulting from ingestion of a common food. We included an outbreak in the study if: (1) chicken liver or a food containing chicken liver (e.g., chicken liver pâté) was implicated; and (2) at least one of the case-patients in the outbreak had a laboratory-confirmed foodborne infection. We described outbreaks by several characteristics, including illness-onset dates; etiologies; case-patient demographics; reported illnesses, hospitalizations, and deaths; implicated food vehicles; food-preparation settings; and contributing factors. For outbreaks with incomplete information about certain characteristics, we attempted to obtain the information from relevant state and local health departments. We compared median numbers of illnesses per outbreak using the Wilcoxon rank-sum test in SAS version 9.4 (SAS Institute, Cary, NC).

## Results

**FIG. 1.**
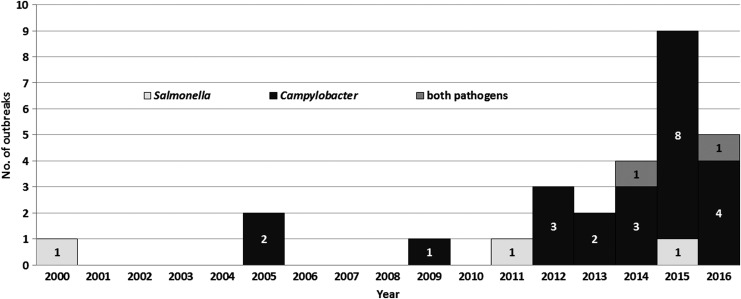
Chicken liver–associated campylobacteriosis and salmonellosis outbreaks (*n* = 28) by year, United States, 2000–2016.

**FIG. 2.**
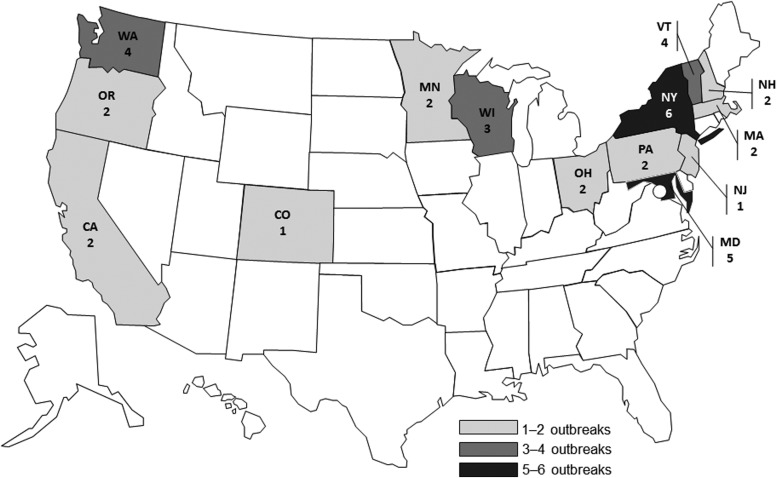
Chicken liver–associated campylobacteriosis and salmonellosis outbreaks (*n* = 28) by state of case-patient residence, United States, 2000–2016 (Four outbreaks included case-patients from multiple states).

During 2000–2016, a total of 28 reported outbreaks associated with chicken liver were identified (Table 1). The frequency of identified outbreaks increased throughout the study period, particularly during its last few years (Fig. 1); for example, there were 4 (14.3%) outbreaks during 2000–2010, 6 (21.4%) during 2011–2013, and 18 (64.3%) during 2014–2016. The outbreaks were clustered geographically, by state of case-patient residence, in northeastern, western, and upper midwestern states (Fig. 2).

**Table 1.** Characteristics of Chicken Liver–Associated Campylobacteriosis and Salmonellosis Outbreaks (*n* = 28), United States, 2000–2016

*Sequential No.*	*Illness onset date range*	*Etiology(ies)*	*State(s) with illness*	*No. of illnesses*^a^	*No. of hospitalizations*^b^	*Implicated chicken liver product(s)*	*Food preparation setting(s)*	*Contributing factor(s)*^c^	*Data source(s)*
1	August 2000	*S.* Heidelberg	CA	4	1	Chicken liver (specific dish/preparation method unknown)	Private residence	None identified	FDOSS
2	August 2005	*Campylobacter* (NOS)	WA	9	0	Chicken liver pâté	Restaurant (sit-down)	Inadequate cooking	FDOSS
3	October 2005	*Campylobacter jejuni*	WI	13	2	Chicken liver pâté	Religious facility	Inadequate cooking; food handler was positive for *C. jejuni*	FDOSS
4	August 2009	*C. jejuni*	CA	6	0	Whole chicken livers	Restaurant (sit-down)	Inadequate cooking/not cooking	FDOSS
5	April to November 2011	*S.* Heidelberg	NY, NJ, PA, MD, OH, MN	190	30	Whole chicken livers; chicken liver spread	Grocery store; school; private residence; assisted living facility	Inadequate cooking; potentially misleading labeling and product appearance^d^	FSIS; FDOSS; published report 1^e^
6	April to May 2012	*C. jejuni* and *Campylobacter coli*	MD, NH	11	2	Chicken liver pâté	Restaurant (sit-down)	Inadequate cooking	FDOSS
7	April to September 2012	*C. jejuni*	VT, NH, NY	6	2	Chicken liver mousse	Restaurant (sit-down); private residence	Inadequate cooking; occupational exposure at poultry slaughter establishment	FSIS; FDOSS; published report 2^f^
8	October 2012	*C. jejuni*	OR	9	0	Chicken liver pâté	Private residence	Inadequate cooking	FSIS; FDOSS
9	April 2013	*C. jejuni*	NY	20	0	Chicken liver spread	Restaurant (sit-down)	Inadequate cooking	FDOSS
10	December 2013 to January 2014	*C. jejuni*	OR, OH, WA	6	0	Chicken liver pâté; raw, pill-sized chicken liver pieces^g^	Restaurant (sit-down)	Inadequate cooking	FSIS; FDOSS; published report 3^h^
11	May to July 2014	*C. jejuni* and *C. coli*	MD	4	0	Chicken liver pâté	Restaurant (sit-down)	Inadequate cooking	FDOSS
12	August 2014	*C. jejuni* and *S.* Enteritidis	MA	5^i^	0	Chicken liver pâté	Restaurant (sit-down)	Inadequate cooking; food handler was positive for *Campylobacter* (NOS)	FDOSS
13	September to December 2014	*C. jejuni* and *C. coli*	VT	4	4	Chicken liver pâté/mousse	Assisted living facility	Inadequate cooking	FDOSS; FSIS
14	December 2014	*C. jejuni*	PA	3	0	Chicken liver pâté	Private residence	None identified	FDOSS
15	March to April 2015	*C. jejuni*	WA	2	2	Chicken liver pâté	Restaurant (sit-down)	Inadequate cooking	FDOSS
16	April 2015	*S.* Enteritidis	NY	7	1	Chicken liver butter	Restaurant (sit-down)	Inadequate cooking	FDOSS
17	May to July 2015	*C. jejuni* and *C. coli*	MN	9	0	Chicken liver mousse	Restaurant (sit-down)	Inadequate cooking	FDOSS
18	June 2015	*C. jejuni*	MD	7	1	Chicken liver mousse	Restaurant (sit-down)	Inadequate cooking	FDOSS
19	July 2015	*C. jejuni*	NY	2	0	Seared chicken liver	Restaurant (sit-down)	Inadequate cooking	FDOSS
20	August 2015	*C. jejuni*	MD	2	0	Whole, fried chicken livers	Restaurant (sit-down)	Inadequate cooking	FDOSS
21	October 2015	*C. jejuni* and *C. coli*	WI	7	0	Chicken liver pâté/mousse	Restaurant (sit-down)	Inadequate cooking	FSIS
22	November 2015	*C. jejuni*	VT	3	0	Chicken liver mousse	Restaurant (sit-down)	Inadequate cooking	FDOSS
23	December 2015	*Campylobacter* (NOS)	CO	4	0	Chicken liver pâté	Restaurant (sit-down)	Inadequate cooking	FDOSS
24	January 2016	*C. jejuni*	VT	5	0	Chicken liver mousse	Restaurant (sit-down)	Inadequate cooking	FDOSS; FSIS
25	April to May 2016	*C. coli* and *S.* Enteritidis	WI	9^j^	0	Chicken liver pâté	Restaurant (sit-down)	Inadequate cooking	FDOSS
26	July 2016	*C. jejuni*	WA	5	0	Chicken liver mousse	Restaurant (sit-down)	Inadequate cooking; ill food handler	FSIS; published report 4^k^
27	August 2016	*C. jejuni*	NY	4	0	Chicken liver mousse	Restaurant (sit-down)	Inadequate cooking	FDOSS
28	September 2016	*C. jejuni*	MA	5	1	Chicken liver pâté	Banquet facility (for senior citizen housing)	Inadequate cooking	FDOSS

^a^ Includes confirmed and probable/suspect case-patients.

^b^ Hospitalization status was not reported for each case-patient. No deaths associated with these outbreaks were reported.

^c^ Not all potential contributing factors were known or listed.

^d^ Product was labeled “broiled” and appeared, but was not, fully cooked.

^e^ Published report 1: Hanson *et al.* (2014).

^f^ Published report 2: Tompkins *et al.* (2013).

^g^ One case-patient in this outbreak consumed raw, pill-sized pieces of chicken liver that had been prescribed by a naturopathic physician.

^h^ Published report 3: Scott *et al.* (2015).

^i^ In this outbreak, *C. jejuni*, *S. enterica* serovar Enteritidis, or both, respectively, were isolated from two, one, and two case-patients.

^j^ In this outbreak, both *C. coli* and *S.* Enteritidis were isolated from each of the four confirmed case-patients (the other five were probable illnesses).

^k^ Published report 4: Glashower *et al.* (2017).

FDOSS, Foodborne Disease Outbreak Surveillance System (Atlanta, GA: U.S. Department of Health and Human Services, Centers for Disease Control and Prevention; data received on December 8, 2017); FSIS, United States Department of Agriculture, Food Safety and Inspection Service; NOS, not otherwise specified.

The 28 outbreaks resulted in a total of 361 illnesses, 46 (12.7%) hospitalizations, and no deaths. The median number of illnesses per outbreak was 5.5 overall (range, 2–190). In general, later outbreaks had fewer illnesses per outbreak; for example, the median number of illnesses per outbreak significantly decreased from 9 during 2000–2013 to 4.5 during 2014–2016 (*p* = 0.002). Among case-patients with available information, 177 (51.2%) of 343 were women and 196 (62.6%) of 313 were ≥20 years of age.

Of the 28 outbreaks, 23 (82.1%) were caused by *Campylobacter* only and 3 (10.7%) by *Salmonella* only; in 2 (7.1%) of the outbreaks, both pathogens caused illnesses. Chicken liver pâté or other blended dishes (e.g., spread, mousse, or butter) were implicated in 24 (85.7%). The implicated chicken liver was reported to be inadequately cooked (or raw) in 26 (92.8%). A foodservice venue was identified as a food-preparation setting in 25 (89.3%). Restaurants, specifically sit-down restaurants, were identified in 22 (78.6%) of the 28 total outbreaks and 18 (75%) of the 24 campylobacteriosis outbreaks.

## Discussion

The frequency of restaurants as a food-preparation location observed among outbreaks in this study (78.6% of total and 75% of campylobacteriosis) is higher than that among all foodborne outbreaks of campylobacteriosis (142/451 [31.5%]) and salmonellosis (1039/2297 [45.2%]) reported to FDOSS during 2000–2016 (CDC, 2018). The predominance of foodservice preparation settings in reported chicken liver–associated outbreaks indicates there may be value in targeting restaurants, particularly sit-down restaurants, and other places where chicken liver dishes are commercially prepared for prevention efforts, especially with regard to cooking adequacy. FSIS (2016a) encourages cooking of chicken liver dishes to an internal temperature of 165°F, as measured by a food thermometer, before consumption. Assisted living or senior citizen institutions were noted as food-preparation settings among the outbreaks in this study; special care should be taken to not serve inadequately cooked chicken livers to the elderly or other higher risk populations. Food safety partners from local, state, and federal agencies, academia, and industry should collaborate to develop ways to encourage foodservice workers and consumers to properly cook chicken liver.

Establishments that produce chicken liver for human consumption (e.g., chicken slaughter facilities) should address food safety hazards associated with chicken liver through their Hazard Analysis and Critical Control Points (HACCP) systems. Freezing is an intervention that has been shown to reduce, but not eliminate, *Campylobacter* in chicken liver (Baumgartner *et al.*, 1995; Harrison *et al.*, 2013). Despite potential concerns that freezing may negatively impact palatability, researchers found that consumers in the United Kingdom had an overall sensory preference for chicken liver pâté made from frozen liver versus fresh liver (Hutchinson *et al.*, 2015). In this same study, the researchers demonstrated that organic acid washes reduced *Campylobacter* contamination on chicken liver. High-pressure processing has been shown to reduce pathogens in other chicken products (Solomon and Hoover, 2004; Jackowska-Tracz and Tracz, 2015) and it may also be effective in chicken liver.

To increase the availability of data on chicken liver–specific prevention strategies, In July 2016, FSIS (2016b) included in its list of food safety research priority items specific to chicken liver. Given that inadequate cooking was a common contributing factor among the outbreaks in this study, an important research need is to develop and validate a method of cooking chicken liver, particularly blended chicken liver (e.g., pâté), that is safe to consume and well accepted by both chefs and consumers. FSIS is collaborating with the Agricultural Research Service and universities on chicken liver–related studies to improve understanding of chicken liver contamination with pathogens and of consumer and chef preferences, practices, and knowledge of risk.

Recent FSIS sampling results are consistent with other data demonstrating pathogen contamination in chicken liver. In November 2016, FSIS (2016c) began sampling and analyzing chicken livers from FSIS-regulated establishments for *Campylobacter* and *Salmonella*. For these samples, the livers are rinsed in sterile broth and the rinsate is submitted for analysis. Among chicken liver samples collected by FSIS during November 2016 to November 2017, *Campylobacter* was isolated from 66/87 (75.9%) samples and *Salmonella* from 57/85 (67.1%) samples (FSIS, unpublished data). Although the small sample size precludes estimation of prevalence, these results signal opportunities for improved pathogen reduction in this part of the chicken.

The observed predominance of campylobacteriosis over salmonellosis outbreaks is noteworthy. Both *Campylobacter* and *Salmonella* have been shown to contaminate chicken liver; indeed, two outbreaks in this study involved both pathogens, suggesting concurrent contamination. In addition, both pathogens have been recovered from chicken livers after experimental oral inoculation. Although several published studies have demonstrated the presence of *Campylobacter* in the internal tissues of chicken liver, to our knowledge, no such studies for *Salmonella* have been reported. Although internal *Salmonella* presence may simply not have been assessed, it is also possible that *Campylobacter* could more likely be present than *Salmonella* in internal chicken liver tissues. This would help explain the greater number of campylobacteriosis outbreaks and would further highlight the importance of inadequate cooking as a contributing factor.

The observed increase in reported chicken liver–associated outbreaks, particularly campylobacteriosis, is consistent with a concurrent increase in foodborne campylobacteriosis outbreaks overall (CDC, 2018). One explanation for the increase observed in this study is that these outbreaks could actually have begun to occur more frequently. Alternatively, a number of illness surveillance factors might also have promoted outbreak recognition and reporting in the latter years of the study, including: higher index of suspicion for chicken liver as an illness vehicle after published reports of outbreaks since 2011 (Tompkins *et al.*, 2013; Hanson *et al.*, 2014; Scott *et al.*, 2015; Glashower *et al.*, 2017); the addition of chicken liver exposure to campylobacteriosis questionnaires used in the Foodborne Diseases Active Surveillance Network (FoodNet) in 2015 (CDC, unpublished data); and the addition of campylobacteriosis to the U.S. list of nationally notifiable diseases in 2015 (Adams *et al.*, 2017). In addition, in 2017, CDC added chicken liver to the National Hypothesis Generating Questionnaire for enteric illness outbreak investigation (CDC, 2017b). Over time, these factors may have improved the detection of small campylobacteriosis outbreaks associated with chicken liver that is consistent with both the increase in the number of observed outbreaks and the decrease in median outbreak size over the length of the study period.

The geographic outbreak clustering observed in this study may reflect regional differences in consumption of inadequately cooked chicken liver dishes. Data regarding chicken liver consumption and preparation patterns are currently lacking, but may help focus prevention efforts. Another explanation for the observed geographic differences in number of outbreaks is that, because of variability in *Campylobacter* surveillance, some states may have been more likely to detect and report outbreaks (Geissler *et al.*, 2017). Detection of outbreaks caused by *Salmonella* and other bacterial foodborne pathogens historically has been greatly enhanced through molecular subtyping by pulsed-field gel electrophoresis (PFGE); however, PFGE is not as consistently used to characterize *Campylobacter* because of testing limitations, hampering detection of outbreaks (Gerner-Smidt *et al.*, 2006). As campylobacteriosis became nationally notifiable in the United States only recently, national-level information has not been readily available. Our findings highlight the importance of standardizing national surveillance for campylobacteriosis that is important to understand the burden of illness, identify outbreaks, attribute sources of infection, and target measures for prevention and control.

Illnesses associated with reported outbreaks represent only a small proportion of the true illness burden. According to FoodNet data, <1% and 6%, respectively, of reported *Campylobacter* and *Salmonella* infections are part of a recognized outbreak (CDC, 2014). Furthermore, it is estimated that for every reported case of *Campylobacter* or *Salmonella* infection, there are ∼30 cases undiagnosed or not reported (Scallan *et al.*, 2011). Hence, the actual number of illnesses associated with chicken liver during the study period was likely many times more than the 361 reported here. A case–control study in New Zealand indicated that consumption of chicken liver was a risk factor for sporadic campylobacteriosis (Eberhart-Phillips *et al.*, 1997); to our knowledge, the extent to which chicken liver exposure may be a risk factor for sporadic illness in the United States has not been assessed in a published study.

## Conclusions

The increasing numbers of reported outbreaks in recent years highlight chicken liver as an important foodborne illness vehicle. The consumption of inadequately cooked chicken liver dishes noted in this study poses a food safety concern made greater by the strong evidence suggesting the presence of pathogens inside chicken liver. To address this problem, public health officials and stakeholders should collaborate to pursue multipronged prevention strategies, including enhancing illness and outbreak surveillance, pursuing needed research, and encouraging food preparers to fully cook chicken liver dishes.
